# Digital self-help for people experiencing intimate partner violence: a qualitative study on user experiences and needs including people with lived experiences and services providers

**DOI:** 10.1186/s12889-023-16357-5

**Published:** 2023-08-02

**Authors:** Hannah M. Micklitz, Zoë Nagel, Stella Jahn, Sabine Oertelt-Prigione, Gerhard Andersson, Lasse B. Sander

**Affiliations:** 1 Medical Psychology and Medical Sociology, Faculty of Medicine, University of Freiburg, Hebelstraße 29, 79104 Freiburg, Germany; 2 Department of Rehabilitation Psychology and Psychotherapy, Institute of Psychology, University of Freiburg, Freiburg, Germany; 3 Department of Primary and Community Care, Radboud Institute for Health Sciences, Radboud University Medical Center, Nijmegen, the Netherlands; 4 Department of Sex- and Gender-Sensitive Medicine, Medical Faculty OWL, University of Bielefeld, Bielefeld, Germany; 5 Department of Behavioural Sciences and Learning, Linköping University, Linköping, Sweden; 6 Department of Biomedical and Clinical Sciences, Linköping University, Linköping, Sweden; 7 Department of Clinical Neuroscience, Karolinska Institute, Stockholm, Sweden

**Keywords:** Intimate partner violence, Digital intervention, E-Health, Virtual-delivery of interventions, Integrative intervention, Advocacy-based intervention, Psychological intervention, Trauma-sensitive design

## Abstract

**Background:**

Intimate partner violence (IPV) is a prevalent public health issue associated with multiple physical and mental health consequences for survivors. Digital interventions can provide low-threshold support to those experiencing IPV, but existing digital interventions have limited efficacy in improving the safety and mental health of IPV survivors. Digitally adapting an integrative intervention with advocacy-based and psychological content holds promise for increasing the efficacy of digital interventions in the context of IPV.

**Methods:**

This study examines the needs, acceptability and usability of an integrative digital intervention for people affected by IPV. We used the think-aloud method and semi-structured interviews with a sample of six people with lived experiences of IPV and six service providers. We analyzed the data using thematic analysis.

**Results:**

We identified the increasing general acceptance of digital support tools and the limited capacity of the current support system as societal context factors influencing the acceptance of and needs regarding digital interventions in the context of IPV. An integrative digital self-help intervention offers several opportunities to complement the current support system and to meet the needs of people affected by IPV, including the reduction of social isolation, a space for self-reflection and coping strategies to alleviate the situation. However, potentially ongoing violence, varying stages of awareness and psychological capacities, and as well as the diversity of IPV survivors make it challenging to develop a digital intervention suitable for the target group. We received feedback on the content of the intervention and identified design features required for intervention usability.

**Conclusion:**

An integrative digital self-help approach, with appropriate security measures and trauma-informed design, has the potential to provide well-accepted, comprehensive and continuous psychosocial support to people experiencing IPV. A multi-modular intervention that covers different topics and can be personalized to individual user needs could address the diversity of the target population. Providing guidance for the digital intervention is critical to spontaneously address individual needs. Further research is needed to evaluate the efficacy of an integrative digital self-help intervention and to explore its feasibility it in different settings and populations.

**Supplementary Information:**

The online version contains supplementary material available at 10.1186/s12889-023-16357-5.

## Background

Intimate partner violence (IPV) represents a significant public health challenge that affects individuals worldwide [1]. Research indicates that IPV against women has a global lifetime prevalence of approximately 26% [95% confidence interval (CI) = 22% to 30%]), making IPV the most common form of violence against women [1]. Nevertheless, men and non-binary individuals, too, are affected by IPV at high rates [2–4]. Experiencing IPV is associated with adverse physical [5] and mental health outcomes [6–9] as well as negative impacts on the social, academic and economic situation of survivors [9–11] and affected children [12–14]. As a result, many IPV survivors express a need for comprehensive psychosocial support to improve their safety and to cope with various mental health-related and socioeconomic challenges [15–18].

Professional psychosocial interventions to support survivors of IPV include advocacy-based and psychological interventions [19, 20], which can be combined into integrative or multi-component approaches [21, 22]. Advocacy-based interventions aim to empower IPV survivors and increase their safety through psychoeducation, risk assessment, safety planning and priority or goal setting exercises [19, 23]. In addition, referrals to specific services are provided to address legal, financial and health needs [19, 23]. Psychological interventions aim to address the psychological consequences of IPV and typically involve training in cognitive strategies, emotion regulation, behavioral and/or interpersonal strategies [24, 25]. Integrative or multicomponent interventions combine the outlined components of advocacy-based and psychological interventions to address the multiple psychosocial needs of people experiencing IPV [21, 26]. In addition to their approach and focus, psychosocial interventions vary in their intensity, setting and service provider [22]. Systematic reviews and meta-analyses suggest that especially integrative interventions with higher intensity have positive effects on the safety, mental health and psychosocial well-being of survivors [21, 22, 26].

Unfortunately, many people who experience IPV remain without professional help [27, 28]. There are several structural barriers that hinder access to professional help, such as limited availability or capacity of support services [28–30]. In addition, several individual barriers impede successful help-seeking, including the lack of knowledge about professional support options, privacy concerns, fears about the consequences of seeking help, beliefs that IPV is a personal problem, or that help is unnecessary, a lack of social support, logistical barriers, financial barriers, as well as language and cultural barriers [28, 30, 31].

In an effort to address these barriers, and encouraged by the challenges posed by the COVID-19 pandemic, research has increasingly focused on digital self-help support interventions for IPV survivors [32–37]. Digital self-help interventions can provide support anonymously, around the clock and offer easy access [33]. Among the most thoroughly researched digital interventions for IPV are safety decision aids (SDAs) [36–38]. Using an advocacy-based approach, SDAs provide safety planning tailored to the user’s acute danger level and safety priorities (e.g., commitment to relationship, resources, own or children’s well-being) [39–43]. They also provide options for action and information about available services and related topics (e.g., physical and mental health, healthy relationships, warning signs) [44]. Other advocacy-based digital interventions provide brief motivational and educational videos for specific populations [45, 46]. Self-help forums have been implemented digitally to increase social connectedness [47, 48]. Digital psychological treatments have been developed for women with prior IPV experiences and acute symptoms of PTSD and depression [49, 50]. Preliminary evidence suggests that digital interventions are highly accepted and are generally considered to be helpful and safe by IPV survivors [35, 51]. However, meta-analyses have indicated that existing digital interventions have no effects in reducing the recurrence of IPV (SMD -0.01 [95% CI = -0.11, 0.08]), symptoms of depression (SMD -0.13 [95% CI = -0.37, 0.11]) and PTSD (SMD -0.11 [95% CI = -1.04, 0.82] in IPV survivors compared to controls [26, 38].

Based on the outlined positive evidence of higher intensity integrative interventions identified in the face-to-face context [21, 22, 26], we are currently developing a more comprehensive integrative digital self-help intervention for individuals affected by IPV. In this study, we include people with lived experiences into intervention development, which has established as a critical step to gain an authentic and in-depth understanding of the psychosocial context, preferences and needs of the target group regarding digital support [35, 37, 52]. Additionally, we include psychosocial service providers to receive insights into the current support system, and the steps and challenges in the support process [22, 37, 44].

In addition to semi-structured interviews, we use the think-aloud method, which is known from other fields to be particularly useful for exploring users’ experiences with digital interventions in an in-depth and nuanced way [52]. In addition to exploring appropriate intervention content, this method also assesses the suitability of the intervention design. Design aspects such as the user interface, navigation, language, and visual aids can significantly influence the user experience and may be critical to the intervention acceptability, uptake and retention [53]. However, such design aspects have received limited attention in qualitative research on digital interventions for IPV survivors so far [37, 54].

## Methods

This study uses the think-aloud method and semi-structured interviews with the aim to explore the acceptability, usability, and needs of people with lived experiences of IPV and service providers regarding a digital integrative self-help intervention. We report this study according to the Standards for Reporting Qualitative Research (SRQR; [55]) and adhered to the items of the CASP Qualitative Checklist [56].

### Researcher characteristics

The research team consisted of one Ph.D. student (HMM, M.Sc. in Clinical Psychology) and two graduate students (ZN and SJ, both B.Sc. in Psychology and in the final year of their Masters in Clinical Psychology). The team was supervised by LBS, a licensed psychotherapist and associate researcher, and received input and feedback from senior researchers (SOP, GA), who have extensive research experience in this area. The study was conducted in collaboration with a professional IPV agency (*Freiburger Fachstelle gegen Häusliche Gewalt*), which supported the study design and recruitment. An employee of this agency participated in the study as a service provider. We presented the study analyses and results at a conference of this agency, attended by experts and people with lived experiences to obtain their feedback and to increase the credibility of the findings. We have reflected on and acknowledged the researchers influence in the research process including data collection and analysis.

### Study recruitment and population

We distributed a flyer with information about the study via existing mailing lists of professional support networks and in self-help forums and groups. For people with lived experiences of IPV, inclusion criteria were a) fluency in German, b) a minimum age of 18 years and c) self-reported past experience of any form of IPV. For the professionals, inclusion criteria were a) fluency in German, b) a minimum age of 18 years and c) work experience with survivors of IPV. All individuals who met these inclusion criteria, were interested in study participation and gave consent were included. We stopped recruitment when saturation was reached, which occurred when the research team agreed that no new themes emerged during data collection.

This resulted in a sample of six individuals with lived experiences of IPV and six professionals who provide psychosocial care to IPV survivors. Table 1 shows the characteristics of the participants. All six IPV survivors identified as female and heterosexual. Their ages ranged from 22 to 54 years (*M* = 39.00, *SD* = 12.51). All but one of the participants held a university degree and were currently employed. In terms of their IPV experience, all except for one participant had experienced multiple forms of IPV, including physical and psychological violence. Of the six IPV survivors, five had received informal support (i.e., from family or friends), and four had received professional support (i.e., police, general practitioner, counseling, couple therapy, psychotherapy) related to their IPV experience. All participants reported using the Internet at least once a day.

**Table 1:** Participant characteristics

**Participant**	**Age**	**Children**	**Type of IPV experienced**	**Support sources used**	**Completed module**
S1	26	No	phy; psy; sex; eco; dig	fri	Self-esteem
S2	48	No	phy; psy	fam; psychoth; face-to-face self-help	Self-esteem
S3	42	Yes	phy; psy; eco; dig	fam; pol; gp; counsel face-to-face, via tel and chat; couple, online self-help	Psychol. cons
S4	54	Yes	phy; psy; sex; eco; dig	fam; pol; gp; counsel face-to-face; psychoth; online self-help	Psychol. cons
S5	42	Yes	phy; psy; sex; eco; dig; stalk	fam; fri; pol; gp; counsel face-to-face; couple; psychoth, online self-help	Self-esteem
S6	22	No	sex	none	Psychol. cons
**Participant**	**Age**	**Children**	**IPV-related work experience in years**	**Profession**	**Completed module**
E1	38	No	12	Psychologist	Self-esteem
E2	36	No	1.5	Social worker	Psychol. cons
E3	63	Yes	25	Social worker	Psychol. cons
E4	26	No	2	Social worker	Psychol. cons
E5	49	Yes	11	Psychologist	Self-esteem
E6	48	Yes	12	Psychologist	Psychol. cons

*Abbreviations*: *phy* physical violence, *psy* psychological violence, *sex* sexual violence, *eco* economic violence, *dig* digital violence, *stalk* stalking, *fri* friends, *fam* family, *pol* police, *gp* general practitioner; counsel = IPV-related counseling; face-to-face = face-to-face; tel = telephone; couple = couple counseling; psychoth = psychotherapy; psychol. cons. = psychological consequences

The six IPV experts included in the study identified as female and heterosexual. Their ages ranged from 26 to 63 years (*M* = 43.33, *SD* = 12.83). All professionals held a university degree and were currently employed. Three professionals were social workers and three were psychologists. One of them worked as an outpatient clinical psychotherapist, and the other experts worked in institutions specialized in IPV or related topics. The length of their working experience with IPV survivors ranged from 1.5 years to 25 years (*M* = 10.58, *SD* = 8.58). None of the experts reported having experienced IPV themselves. All but one IPV expert reported using the Internet at least once a day.

### Ethical considerations

The Ethics Committee of the University of Freiburg, Germany, approved the study procedures (No. 21–1593). We informed the study participants about the objectives of the study, the study procedures and data protection. All participants gave voluntary informed written consent to participate and were informed of their right to withdraw consent at any time, to skip uncomfortable questions, and to take breaks.

Participants with lived IPV experiences were offered comprehensive written information about medical and psychosocial support services. If they expressed, or we suspected, psychological distress during study participation, we offered an immediate conversation with a licensed psychotherapist. Furthermore, we offered them contact referral to an outpatient psychotherapy clinic. However, no participant requested psychological support or contact referral. Participants with lived experienced of IPV received a gift voucher of €50 as compensation for their participation.

The data records were stored on a secure server at the University of Freiburg, Germany, and were accessible only to authorized project staff who were bound by confidentiality.

### Intervention

We conducted this study as part of the development process of an integrative digital self-help intervention for people experiencing IPV (*by my side)*. For the purposes of this study, we presented one of two sample modules to the study participants. Sample module 1 focused on the topic of the psychological impact of IPV and available support services, representing an advocacy-based intervention component. Sample module 2 had a more psychotherapeutic approach and addressed self-esteem. Table 2 shows the content of the sample modules.

**Table 2:** Content of sample of modules

Module	Content
Psychological consequences of IPV	Psychoeducation on mental health disorders and the psychological consequences of IPV, illustrating case examples, symptom check, information on available support services
Self-esteem	Psychoeducation on the self-esteem and the impact of IPV experiences on self-esteem, self-reflection-exercise psychosocial factors that influence one’s self-esteem, self-reflection exercise on biases in the perception of one’s self-esteem

### Data collection

We collected the data between January and March 2022. Due to social-distancing restrictions during the COVID-19 pandemic, most interviews were conducted online. The participant and two interviewers attended the session. While the main interviewer guided through the procedures and led the interview (ZN or SJ), the secondary interviewer (HMM) observed, took notes, asked supplementary questions and intervened in case of uncertainties. We provided participants with information about the study’s aim and procedures. Participants gave written informed consent and completed questionnaires on their demographics, digital literacy and IPV experience or work experience, respectively, using an online form [57].

In the first part of the qualitative data collection, we used the think-aloud method, in which participants worked through a sample module and vocalized all of their impressions, thoughts and feelings [58]. Participants received detailed instructions on the method and if necessary, think-aloud prompts while working through the module (e.g., “Feel free to talk more about what you think or feel about this!”). In the second part, we conducted semi-structured interviews to further elaborate on the users’ experience, acceptance and needs regarding the intervention. The interview questions were related to the content, design and language of the sample modules (e.g., “How do you evaluate the content of the module presented?”) as well as the participants’ needs, prior experiences and opinions regarding digital interventions (e.g., “Have you ever sought support on the Internet? Tell me about it.”). Supplement A provides an English translation of the interview guide. We recorded both the think-aloud sessions and interviews in audio format, ranging in length from 36 to 61 min.

### Data processing and analysis

We transcribed the audiotapes verbatim using *f4* software [59]. We removed text passages that could identify the research participants. We analyzed the transcripts using *MAXQDA* software [60]. The transcripts of the think aloud sessions and the interviews were analyzed together.

We used Kuckartz’ thematic analysis (also known as qualitative content analysis) [61]. We were guided by an interpretative phenomenological approach with a focus on understanding the subjective lived experiences of the participants, interpreted in the context of broader systems and in relation to existing literature [62]. Our goal was to develop a category system consisting of main and subcategories that broadly represented multiple views and summarized commonalities as well as disagreements among participants. We developed the category system deductively and inductively in an iterative process. Following Kuckartz [61], we first closely read through the transcripts to familiarize ourselves with the data and to identify relevant themes, which we captured in memos. We constructed main categories based on the memos, existing literature and our research questions. We then sequentially analyzed the transcripts and assigned relevant text passages to the corresponding main categories. Based on these text segments, we developed subcategories. ZN conducted this procedure for the data collected from participants with lived IPV experiences, while SJ analyzed the data from the expert sample. They both worked independently from each other, but met throughout the process to discuss the analyses together with HMM to increase intersubjectivity. HMM reviewed the two resulting category systems, integrated them into one final category system, and coded the entire interview material based on this category system. The views of both groups were given equal weight. Unless otherwise indicated, they are reflected equally in the analysis. We have selected the following quotes to substantiate and illustrate our findings, regardless of the participant’s group affiliation.

## Results

Our analyses resulted in a category system consisting of the following 8 main categories: societal context factors, previous use of digital supports, application possibilities, perceived barriers, perceived limitations, target group characteristics, intervention design features and intervention content. Each main category further includes 2 to 8 first-level subcategories and 3 to 15 s-level subcategories. Findings on previous use of digital supports are presented in Supplement B. The remaining results are presented below.

### Societal context factors

Participants identified societal contextual factors that influence the acceptance of and need for digital support measures for individuals experiencing IPV. Specifically, service providers reported an increasing acceptance of digital services, not only due to the COVID-19 pandemic, but also due to a general societal shift toward digitalization.

“Society is already more open to online programs and I can well imagine that everything is being digitalized, so I think this has great potential for the care system.” (E2)

Another contextual factor mentioned was the limited capacity of the current support system, particularly the limited access to psychotherapy, which concerned people with lived experiences and service providers alike.

“There are counseling services for IPV, yes. A doctor, general practitioner, yes, I was seeking help there. But psychotherapists, I thought you’re joking, because it’s a catastrophe. You won’t even get on a waiting list.” (S3)

“This [*by my side*] would be something, I’d really like to recommend to my clients, because I would know, it provides useful support, is easily refundable by health insurance, of course I’d recommend this. I think it would be an enormous relief, especially for clients, where I’m afraid that they won’t even get on a waiting list for psychotherapy.” (E4)

Participants agreed that digital interventions should not replace face-to-face services and cannot be relied on alone to address the limited capacity of the current support system. However, they acknowledged the potential benefits of digital interventions and identified several ways in which they could be integrated into the existing support system.

### Applications of digital interventions within the current support system

The majority of participants expressed the need for digital interventions as a low-threshold entry point to the support system to reduce social isolation.

“You are feeling so isolated. And then you start looking for help options, but you can’t find anything. Or you only find help options like shelters and think ‘oh, my experience wasn’t that bad. These are women with children, they are really afraid of their partners, they need to get themselves to safety. I can’t show up there’. That’s why I think such an intervention is so good for these people, who are feeling isolated, but don’t know where to go to. They need something low-threshold.” (S1)

Participants emphasized that digital interventions should ideally serve as a bridge over to face-to-face psychosocial support, but acknowledged the limited availability of such support. As a result, they emphasized the need for digital interventions to provide longer-term psychological support, a room for self-reflection and coping strategies.

“I thought, the goal of the intervention should be to show how to handle the violence. Because […] psychotherapy or counseling is not accessible in a lot of cases. That’s why I had expected that it [*by my side*] also provides coping strategies in case I can’t access further help services. So, I thought, the goal is not only to say “here you can get help”, but to provide coping strategies within this digital format.” (S6)

“My biggest concern is: is it only this one module or does it have several modules? So that, once you made a step forward, you are not by yourself again, but you can continue [your path].” (S4)

Participants with lived IPV experiences reported a need for help in situations of conflict or when feeling overwhelmed or distressed. The participants indicated that after receiving in-person services, the digital intervention could be valuable to provide blended therapy.

“When I experience acute conflicts, of course, right in this moment, you can’t be on your phone, but right after I’d […] like to process this situation. For example, […] there could be suggestions on how to resolve the conflict, or input on, what I can do right know to calm down.” (S2)

“You can access it [*by my side*] 24/7. The self-help-group is once a month, psychotherapy once a week. On the one hand, to me that is almost too often. On the other hand, it is never in the situation you urgently need advice. That’s why I think, a digital solution is a great […] addition.” (S3)

Despite the potential benefits of digital interventions for people experiencing IPV, participants identified several barriers that need to be considered when implementing such interventions. Some of these barriers were specific to digital interventions, including the need for linguistic and digital literacy, as well as access to a web-enabled PC or smartphone. Experts were particularly concerned about the limited ability of digital interventions to spontaneously adapt to specific needs and provide emergency interventions. In addition, both experts and people with lived experiences of IPV agreed that a stand-alone digital intervention is not sufficient for treatment and lacks the personal interaction of face-to-face support. Some participants also expressed concern that the use of a digital intervention could discourage survivors from seeking further face-to-face services.

Other barriers were related to the life circumstances and hardships faced by people experiencing IPV, which need to be considered when developing digital self-help interventions for this population.

### Target group characteristics

#### Capacities

As one relevant barrier, the psychological well-being and capacities of the target group were mentioned. People with lived experiences reported on the psychological distress they experience as a result of IPV.

“I forget a lot of things, I’m disoriented. Of course, if you’re awake the whole night, of course you’re disoriented.” (S3)

The psychological distress resulting from IPV, combined with everyday stressors, may interfere with the target group's ability to fully engage in and benefit from the intervention.

“It could be too much. Multiple stressors, but it’s the same for ‘normal’ therapy. When I think about a single mom with three kids, how would she manage to take the time and calmness to work through this properly.” (E4)

“It offers a lot of things: I can reflect about me and my situation, how I’m doing, I get a lot of information. […] It’s really good, that it’s so extensive, and that it provides all the relevant links and telephone numbers. But it’s nothing you can do on top [of your everyday life]. You need to resolve this conflict: it needs to be suitable for everyday life, you can work through it on the side, maybe even on your smartphone. Yet at the same time, it requires a lot, it is exhausting to think about yourself and to get into this work.” (E4)

Furthermore, participants expressed that working through the intervention could potentially cause psychological distress, for example if the content was too extensive or demanding, if the wording was too explicit, or if the prefabricated content did not match the user’s personal experience.

“I don’t know if they [survivors of IPV] could handle this […] tool, or if they would break down and think ‘I’m a failure, why should I write anything here?’” (S5)

#### IPV experience

The persistence, form, and intensity of the violence experienced emerged as another relevant factor. Participants expressed that people experiencing IPV may be prevented from accessing the digital intervention if they lack a private and safe space, especially those who are acutely experiencing IPV. In addition, participants raised concerns about the safety of clients if the perpetrator found out about the intervention.

“Of course, it can be dangerous, when I work through [this intervention] and my partner enters the room and sees what I’m doing. But I think that doesn’t speak against this support option, it rather reflects the reality of violence.” (E3)

“If your partner controls everything, which is often the case in these relationships, when he tracks the browser and checks, which websites I have accessed. Of course, it is difficult. But it is also difficult to call somewhere, to make an appointment with a counseling service. That’s the point, when I’m locked up in a domestic control situation and my partner controls everything, then I don’t have a chance to get access to any service […], no matter which services you provide.” (E3)

Participants mentioned that the intervention needs to take into account the form and severity of IPV and the level of danger. This information needs to be assessed, considered, and effectively communicated within the intervention.

“Some, they are insulted, but there is no acute threat. Yet, they suffer a lot. It’s also alarming. However, others, they are highly and acutely threatened, they get death threats, but they don’t realize how dangerous it is, and I could imagine that some people in this situation would think ‘oh, […] that’s interesting’ […], when actually something else is indicated.” (E4)

At the same time, participants expressed concern that downplaying the seriousness of psychological IPV or "milder" forms of physical IPV could discourage people experiencing these forms of violence from using the intervention.

Participants noted that some of these concerns are only relevant for people who remain in the violent relationship, and therefore, it is important to consider whether the target user is separated.

“You need to differentiate in which situation the person is. Are they freshly separated? Not yet separated? Longer separated? Or separated for the fifth or seventh time?“ (S5)

#### Stage of awareness

Related to this, participants considered the user's stage of awareness as relevant to the aim, content, and wording of the intervention. They emphasized the necessity of identifying as someone affected by IPV and in need of help as a barrier to accessing this support option.

“A relevant point is [identifying as someone who is affected by IPV], but they won’t go to counselling services either. It’s about confronting yourself with your own misery and pain.” (E3)

Participants indicated that for people at an earlier stage of awareness of their own IPV experience, the intervention should provide a space for self-reflection and to receive information on IPV.

“It should be one aim of the intervention, to figure out ‘am I affected by violence?’ and it [the intervention] should support you during that process. Because, I think, otherwise, I wouldn’t have thought, that it [the program] actually addresses me.” (S6)

For users at this earlier stage of awareness, some of the content provided may be too demanding and distressing, as it may take a considerable amount of time to fully grasp what is happening and to understand the causes and consequences of IPV.

“Wow, you are assuming a lot here. […] that’s a very, very long process of self-reflection […]. I think it took me months to understand this. I think if you have experienced violence and you are at this stage of self-reflection, then you’re really progressed. I don’t know, if you haven’t gone through this process of self-reflection, and you hear this sentence, wow, difficult.” (S5)

Participants expressed differing opinions about the usefulness of the intervention for individuals who have progressed in their self-reflection process.

“I think that it is probably a good tool for people, who are already out of the situation, who have worked through some things already.” (S1)

“I think, women who know words like ‘gaslighting’, they already worked on these topics intensely, they probably wouldn’t need an online tool anymore.” (E6)

Participants requested that the intervention should be tailored to a specific target group, which should be clearly defined to ensure that the intervention meets the needs and expectations of the intended users.

“I think, it’s important to separate the stages that someone goes through and then make individual offers to the individual stages.” (S1)

“I basically think that for people who are actually in the situation, this word ‘violence’ or anything, is just what makes one shy away a little bit. I think, for people in this situation it would be more a wording like [...]: ‘Have you ever felt unsafe?’ [...] I think it helped me a lot to mention this feeling of fear or danger, because I was always a bit afraid of him. And I think something like that could be helpful to get started. But I really think that when the person is out of the situation and a little bit of time has passed or something like that, it’s actually good to hear: ‘Hey, you've been wronged’, sort of. I think I would in fact find it rather annoying if the perpetrator is not referred to as the perpetrator [...]." (S6)

However, the participants were ambivalent about when certain content would be beneficial and when it could lead to distress.

“On the one hand, […] a lot of them [survivors] are so distressed, it’s an acute conflict, I think it could be too much information for them. On the other hand, especially for them it’s so relevant that they get [this information on help options], that they are ask ‘how are you doing?’ Thinking about their own psychological symptoms, that’s the questions, is it good to do this right at the beginning, or is that something to do as a second or third step?” (E4)

“Thinking about your self-esteem, that’s something it would start, hm, it’s difficult, probably after separation. But on the other hand, it could help you to find the energy to go through the separation” (S5)

Furthermore, participants pointed out the diversity of the target group, which poses a challenge in defining distinct target groups with specific needs.

“Some clients, they need you to name it, so they take themselves seriously, because they can’t name it and they downplay it and they get stuck with this, but for other clients, you need to be cautious, so it’s not triggering. I think it has individual effects. Some may need, that you name it, that what happened was violence. But you have to detangle it before, how you define violence, especially psychological violence.” (E6)

Survivors are so diverse. There is no ‘one-fits-all’-solution for intimate partner violence. It’s not one certain disorder, but one survivor is super resilient, she receives practical help for one week and doesn’t suffer any psychological consequence. Another survivor is highly traumatized, another survivor suffers from anxiety, so their needs are very diverse.” (E4)

### Intervention content

Participants also expressed divergent opinions regarding the different components of the digital intervention. While they appreciated the empathy, support, and practical tips provided, they had differing opinions about the scope of the psychoeducation and self-reflection exercises as well as the helpfulness of the case examples. The participants suggested that the digital intervention should cover a range of topics in multiple modules, including basic information about IPV and safety, separation, children, help-seeking, mental health, emotion regulation, self-esteem, posttraumatic growth, and relationships. A detailed presentation of the feedback on the different components and required content can be found in Supplement C.

### Design features

Based on the participants input, we identified several design features that are critical to meeting the diverse needs of the target group while ensuring the usability of the intervention. We provide quotes from participants illustrating these required design features in Supplement D.

In terms of functionality, participants emphasized the need for flexibility, and self-determination while working through the intervention. This included being able to choose the topics they wanted to work on and the desired extent of work they want to do. Participants also requested that the intervention content should be individualized based on their input. Other valued features included an interactive and multimodal design, with clear instructions to make the handling easy. Additionally, safety measures (e.g., an exit button) should be implemented to ensure user safety.

Regarding accessibility, participants highlighted the importance of an anonymous and low-cost intervention, that can also be accessed via smartphone. Additionally, promotion of the intervention was deemed crucial to increase awareness of the existence of this support option, with personal recommendations from fellow survivors or service providers being the preferred method. However, participants also emphasized the importance of public promotion and search engine availability, and suggested employing multiple search terms to increase discoverability.

Inclusive language that addresses target users at different stages should also be considered both, in the public promotion of the intervention and within its content. Furthermore, the diversity of the content was considered relevant, including the representation of people from different cultures and educational backgrounds, people of all genders and with queer sexualities.

Concerning the aesthetics of the intervention, participants valued a clear, neutral, and calming design that promoted a feel-good atmosphere, for example through warm colors, friendly pictures or soothing background music.

Participants appreciated the logical structure of the intervention, including an overview at the beginning of each module that allowed them to anticipate what was coming next. They also appreciated a gentle start to each module.

Regarding the language, many participants pointed out the need for a plain and personal, rather than scientific language. The wording should be clear and direct, while also being differentiated and sensitive, with an appreciative and positive tone. For example, when talking about the psychological effects of IPV, participants mentioned to avoid scientific terms such as ‘vulnerability’ or ‘psychological reactions’, differentiating that the user is not suffering from the psychological symptoms, but from the experience of violence, addressing the user directly with important messages and appreciating the user’s strengths and efforts. Participants liked the use of metaphors and analogies and found them helpful in depicting complex content.

## Discussion

This qualitative study investigated the needs, acceptability and usability of an integrative digital self-help intervention for people affected by IPV with a sample of individuals with lived experiences and service providers. The findings suggest that digital interventions as a support tool for individuals experiencing IPV are well accepted and could complement the current support system in various ways. Space for self-reflection, the reduction of social isolation and the provision of coping strategies to mitigate the situation emerged as central needs of IPV survivors seeking help online, supporting the concept of the integrative self-help intervention. However, limited psychological capacities, potentially ongoing violence and different stages of awareness challenge the development of a digital intervention suitable for the target group. We have received concrete feedback on the components and content of the integrative intervention, and identified design features required for a high intervention usability.

Our participants expressed an increasing acceptance of digital self-help tools due to the general societal shift towards digitalization. Digital interventions are particularly accepted as an easily accessible and privacy-preserving information source and entry point into the support system in the context of IPV [36, 37] and mental health [63, 64]. Consistent with previous qualitative studies in the context of the development of safety decision aids (SDAs), the reduction of social isolation and a room for self-reflection, leading to the recognition of one’s experiences as abuse, emerged as central needs when first seeking help online [44, 65, 66]. Our participants confirmed the need for educational content to raise awareness and elicit change [15]. This includes information on the definition and different forms of IPV, danger assessment, information on related topics (e.g., legal advice, finances and housing) and step-by-step instructions for preparing a separation [44, 65–68]. However, consistent with previous research, we found that these contents can be overwhelming and exacerbate the experienced distress and social isolation [44, 66, 69], if not accompanied by ongoing psychological support. Therefore, digital interventions should ideally serve as a bridge to face-to-face services, that can provide this support [44, 66, 68]. However, our participants pointed out the issue of limited availability of extensive face-to-face support, which digital interventions need to address. While the gaps between service needs and resources are recognized [70, 71], previous digital interventions in the context of IPV have paid limited attention to this problem [37].

As a well-researched treatment measure for mental health disorders [72, 73], self-help interventions hold promise for providing prolonged psychological support digitally. Our participants expressed acceptance of these interventions to address the need for emotional advice and coping strategies to alleviate the situation. Typically, psychological self-help interventions contain multi-modal content that users can read, watch, listen to, and interact with [72]. In this study, we presented sample modules that included common components of such interventions, including psychoeducation, case examples, self-reflection-exercises, practical tips and homework assignments. They were generally considered useful by our participants. In addition to these components, human guidance has positive effects in digital interventions to increase their acceptability, effectiveness and adherence [74–76]. An e-coach can provide human guidance synchronously via chat or telephone or asynchronously via email or by integrating it into the e-health platform. This can include technical guidance (e.g., instructions for resolving technical problems), motivational guidance (i.e., reminders to complete the module), and therapeutic guidance (e.g., clarifying therapeutic content, providing therapeutic feedback on user input, and praising the user) [75]. Additionally, as requested by our participants, an e-coach can address any psychological distress or safety concerns that may arise.

This is particularly important because, in line with previous qualitative research on SDAs, our participants expressed safety concerns when providing digital interventions to people acutely experiencing IPV. Preliminary evidence suggests that the perpetrator’s awareness of digital help-seeking did not lead to an escalation of violence [77]. However, in line with prior research, our participants indicated that it can still be a barrier if the user doesn’t feel safe and calm enough to work through the intervention and properly engage with the content [44, 69]. Building a positive therapeutic alliance is a prerequisite for establishing a sense of safety [22], and can also be achieved in digital interventions [44, 65, 78]. This was confirmed by our participants, who appreciated the empathy, praise, and social support provided by the digital intervention. In addition, the implementation of safety measures (e.g., an exit button; instructions for safe internet use, masking, brainstorming safe places to work through the intervention), along with a strict adherence to safety guidelines is critical to increasing the user safety [69, 77, 79]. Furthermore, potential safety threats and options to increase safety need to be considered and effectively communicated to users [77]. As emphasized by our participants, this has to happen without downplaying the seriousness of psychological IPV or ‘milder’ forms of IPV [44], illustrating the challenge of creating an appropriate intervention that acknowledges the diversity of forms and experiences of IPV.

A related challenge is that potential users may not feel targeted by the wording of the intervention because they do not identify as someone experiencing violence. This can be an initial barrier to seeking help [44, 77], but can also cause distress when working through the intervention. Using less explicit terminology, such as “feeling afraid,” without using the term “violence” might address this issue [44, 77]. However, our participants reported that during the process of self-reflection and change, it is important for some people to explicitly label their experiences as violence. This indicates the need to use inclusive wording to address all stages of self-awareness. Yet, it is unclear whether using this wording throughout the entire intervention is an appropriate solution.

This problem extends beyond the wording and also encompasses the range and depth of topics covered in the intervention. To address different stages of change, some participants suggested defining a clear target group and providing accordingly tailored content. However, other participants pointed out that the needs of the target users are diverse, making it difficult to define a specific target group, which is consistent with prior research [80]. Each recovery process is unique and depends on individual circumstances [16]. While the overall goal should be to achieve safety and improve well-being, IPV survivors should be able to choose their own goals and desired behavior changes [80]. In a digital format, personalization can be achieved by providing multiple modules [50]. The user can select relevant modules based on their individual needs in a shared decision-making process with the e-coach [50]. In this way, a multi-modular digital intervention could offer a unique opportunity to provide ongoing support during the often time-consuming process of realizing and processing IPV experiences, while providing flexibility and self-determination, both of which are critical factors in increasing IPV survivors’ treatment uptake and engagement [22, 81].

Finally, the intervention must consider the psychological capacities of the target group. In addition to everyday stressors, many IPV survivors must cope with safety-related, socioeconomic and legal challenges often associated with IPV [44]. In line with our participants’ experiences, they are also impacted by the neurophysiological and psychological effects of trauma [71], including poor concentration, memory problems, irritability, hypervigilance, and exaggerated self-blame [82, 83]. According to our participants, the intervention could potentially contribute to the distress experienced. Providing trauma-informed care means taking these aspects into account when designing interventions [71]. According to our participants, necessary design features to ensure the intervention usability include the provision of multimodal content, clear, neutral and calming aesthetics that create a feel-good atmosphere, a short logical and predictable structure, a plain and direct, yet appreciative and sensitive language, easy handling and clear instructions, as well as the representation of people from diverse cultures, educational backgrounds, genders and sexualities in intervention content. Additionally, our participants expressed the need for an app-based version of the intervention, as well as cost-free and anonymous access, which is consistent with previous studies on SDAs [44, 66, 68, 69].

While adhering to these design features may improve the acceptance and usability of the intervention, it is important to acknowledge that digital interventions are not without barriers and may exclude users with lower linguistic literacy, disabilities, limited digital literacy or limited access to a PC or smartphone. This is particularly problematic because those who are precluded from using digital interventions largely overlap with generally underserved populations, such as IPV survivors experiencing poverty or homelessness, survivors with disabilities, and survivors from cultural or linguistic minorities [22, 37, 70, 84].

On that note, and turning to the limitations of this study, it should be noted that all of our participants were well-educated, German-speaking and German-born, white, heterosexual, cisgender and mostly able-bodied women, who were willing to participate in research and to share their personal or professional experiences. The selective sample challenges the criterion of saturation, that we used to determine the sample size. Furthermore, the concept of saturation itself has been criticized [85]. Given that this study was part of an intervention development process and had an explorative character, we deemed the outlined procedure and sample size to be appropriate. However, it should be noted that this study does not warrant an exhaustive examination of the targeted subject. For example, it is difficult to generalize the findings to other populations who may have different needs regarding digital interventions [84, 86–88], but also have different experiences of IPV and help-seeking [89–93]. Future research should explore how to adapt the intervention for people from diverse backgrounds, including people with disabilities, people from low- and middle-income countries, marginalized communities in high-income countries, and people from gender and sexual minorities, as well as cisgender men.

Additionally, the study only included psychologists and social workers as service providers, which may have influenced the results. Baird and colleagues [37] have critiqued the underrepresentation of social workers in research on digital interventions in the context of IPV, underscoring the relevance of our findings. However, the inclusion of other stakeholders of the professional IPV support network, such as police officers, policy makers, as well as legal and medical practitioners, could provide additional insights into the needs and challenges faced by those affected by IPV. Survivor professionals, who have both, lived experience and professional knowledge, could contribute another unique perspective [44]. In this context, it might also be interesting to focus the analysis on the divergent views of the different stakeholder groups [44]. However, we decided against this focus of analysis based on our interview data, where such differences did not emerge as central. The perspectives of our participants converged harmoniously and any disagreements arose within the groups rather than between them.

## Conclusion

This study investigated the needs, acceptability, and usability of an integrative digital self-help intervention for survivors of IPV. We used the think-aloud method and semi-structured interviews with a sample of people with lived experiences and service providers. Our findings suggest that an integrative self-help intervention with appropriate safety measures and a trauma-informed design could be a well-accepted complement to the current support system, reducing social isolation and providing space for self-reflection and coping strategies for people affected by IPV. However, the life circumstances and hardships associated with IPV experiences, as well as the diversity of IPV survivors, challenge the development of a digital intervention suitable for the target group. A guided and tailorable multi-modular intervention seems promising to meet the individual needs of IPV survivors. Future research should evaluate the efficacy of a guided integrative digital intervention and explore its usability and feasibility in different settings or populations.

## Supplementary Information

**Additional file 1.**

## Data Availability

The datasets generated and/or analysed during the current study are not publicly available due to the need to protect the confidentiality and privacy of our research participants but de-identified data are available from the corresponding author on reasonable request.

## Abbreviations

ACT: Acceptance and commitment therapy
iCBT: Internet-based cognitive-behavioral therapy
CI: Confidence interval
IPV: Intimate partner violence
M: Mean
PTSD: Posttraumatic stress disorder
SD: Standard deviation
SDA: Safety-decision aid
SMD: Standardized mean difference
SRQR: Standards for reporting qualitative research
