# Supplementary material for: Digital self-help for people experiencing intimate partner violence: a qualitative study on user experiences and needs including people with lived experiences and services providers
Source: BMC Public Health. 2023 Aug 2;23:1471. doi: 10.1186/s12889-023-16357-5 (PMC10394820; doi:10.1186/s12889-023-16357-5)
Supplement: Supplementary file 1 — Additional file 1. [file 12889_2023_16357_MOESM1_ESM.docx]

Supplement

(Digital self-help for people experiencing intimate partner violence: a qualitative study on user experiences and needs of people with lived experiences and services providers, Micklitz et al.)

**Table of content**

[Supplement 1](#_Toc138665409)

[Supplement A: Interview guides 2](#_Toc138665410)

[Supplement B: Previous use and acceptance of digital support measures 4](#_Toc138665411)

[Supplement C: Feedback regarding the different components of *by my side* 4](#_Toc138665412)

[Empathy and support 4](#_Toc138665413)

[Psychoeducation 5](#_Toc138665414)

[Case examples 5](#_Toc138665415)

[Self-reflection exercises 5](#_Toc138665416)

[Practical tips 6](#_Toc138665417)

[Guidance 6](#_Toc138665418)

[Topics 6](#_Toc138665419)

[Supplement D: Required design features 8](#_Toc138665420)

[Functionality 8](#_Toc138665421)

[Aesthetic 8](#_Toc138665422)

[Structure 9](#_Toc138665423)

[Language 9](#_Toc138665424)

[Inclusiveness & Diversity 10](#_Toc138665425)

[Gender 10](#_Toc138665426)

[Sexuality 10](#_Toc138665427)

[Culture 10](#_Toc138665428)

[Education 10](#_Toc138665429)

[Accessibility 10](#_Toc138665430)

## Supplement A: Interview guides

**eTable 1** Translated interview guide

| Topic | Narrative-generating question | Possible follow-up questions |
| --- | --- | --- |
| Contents | How do you evaluate the contents of the presented module? | - Which of the presented components would you work on? - Which of the presented components would you rather not work on? - Which contents did you find were missing in the sample intervention?   *For service providers:*   - Which of the presented components do you (not) consider helpful and why? |
|  | Apart from the module presented, what other topics should be covered in other modules? | - In your opinion, is there a topic that should definitely not be missing in the intervention? - What specific goal should be pursued in the intervention? |
| Specifics implementation/  design/language | How did you perceive the implementation of the intervention? | - What did you find good about the implementation? - What would you do differently? - How did you perceive the language? - Did you find the wording understandable and appropriate? - How did you feel about the design of the intervention? - Any specifics about the technical implementation of the intervention? - How did you find the structure of the sample intervention? - Are there any specifics that should be considered regarding the structure of the intervention? |
| Experience | Have you ever sought support on the Internet? Tell me about it!  *For service providers:*  What role do digital support measures play in your work so far? | - What did you find helpful? - What did you perceive as negative? - What helped you to deal with what you had experienced? - Why has the Internet not been a support option for you so far?   *For service providers:*   - What do you find helpful about it? - What do you perceive as negative? - Why did you not use digital support measures in your work so far? - Can you tell me about situations in which a comparable support measure has/ would have helped your clients? |
| Factors that contribute to take-up  Factors that hinder take-up | Can you tell me something about whether such an intervention would be an option for you to receive professional help?  *For service providers:*  In your opinion, what would contribute to people experiencing IPV making use of this help option?  In your opinion, what would prevent those affected from making use of this help option? | - Why? Why not? - What would motivate you to take up the intervention? - What would make it easier for you to do the modules? - What would cause you to consider this support option? - In what ways or from whom would you like to hear about the intervention? - What would prevent you from using such an intervention? - What would cause you to stop using such an intervention? - What would make you do many of the available modules? - What would you consider very difficult when doing the intervention?   *For service providers:*   - In which situations would such an intervention be helpful for your clients? Why? - When you think about your clients, …   - In what way could the presented intervention be a help? What is important for this?   - What would prevent them from using such an intervention?   - What would cause them to stop using such an intervention?   - What would they find very difficult to deal with during working through the presented intervention? |
| *For service providers only:* Context factors/ Current support system | How do you think about a possible integration of digital interventions into the existing support system? | - Are there any specific characteristics of the German support system, which are relevant in this regard? - What effects would this have on your everyday work? - To what extent could it make your work easier/difficult? |
| - Safety | - Would you feel safe while doing the intervention?   *For service providers:*  How would you rate the safety of the intervention? | - Why? - Why not? - Would you have concerns about your own safety when using such an intervention?   *For service providers:*   - Would you have concerns about the safety of your clients when they use such an intervention? Which ones? |
| - Attitude | - In conclusion, what do you think of this type of support? | - What do you find good about this support option? - What do you consider to be negative about this support option? What concerns would you have? - What should happen in the intervention so that you would have found doing the intervention helpful? - Would you recommend using the intervention to others? Why? Why not? |

## Supplement B: Previous use and acceptance of digital support measures

Most of the service providers had provided counseling services via video-call or telephone themselves. Most services providers reported that they and their clients appreciated the advantages of digital communication, such as increased anonymity and less organizational, time and financial efforts due to the elimination of travel time. One expert was more skeptical, reporting that she and most of her clients preferred direct face-to-face contact. She felt that digital communication led to an increased work load due to technical problems, increased time-effort to answer emails, limited transferability of methods used in face-to-face contact and limited opportunities for personal interactions.

Most people with lived experiences of IPV, too, valued digital support options and had sought some form of digital support before. They had often searched for information about IPV online. One participant had used an online screening tool for PTSD symptoms. Several participants reported using a digital self-help forum for survivors of IPV. One participant had used a digital self-help intervention for stress reduction that helped her identify her relationship as a major stressor in her life. One IPV survivor did not see a digital intervention as a useful support option for her.

## Supplement C: Feedback regarding the different components of *by my side*

### Empathy and support

The participants appreciated the empathy and support provided within the intervention. Especially praise and encouragement, check-ins and reminder to take breaks were perceived positive.

“I’m praised here again, that’s nice for sure. I think it’s so nice that the program somehow notices, that this topic can be challenging and exhausting”. (S5)

“You somehow have to make up for that shortage. You’ve heard so often that you are dumb, that you’re a failure, so it’s good if someone says: ‘Hey, you made a good job here.’ (S6)

### Psychoeducation

While participants agreed on the general relevance of psychoeducation, there were inconsistent needs regarding its scope. Some participants appreciated the extensive information or wanted to read more, others thought it was too much text.

“It’s relevant, if you’ve never thought about it [that topic] before, that you just read something. I think it could be good, to have an emotional distance, and to read through something, a little more neutral so to speak.” (S5)

“I really like knowledge, at least for some topics I’m interested in, I’d like to read more about it, like one page full of information.” (S2)

“I think the reading load, at least to my knowledge, that will be difficult for a lot of people. But I don’t know how to deal with that, because, you won’t succeed [on your journey] without reading.” (S4)

### Case examples

Case examples, too, were perceived differently. While some participants stated not to need them, others thought that they were the most helpful part. Participants could relate to the case examples, which reduced feelings of isolation and provided guidance on how to handle similar situations. Experts valued that the provided case examples reflected the life realities of IPV survivors, while also being moderate enough to avoid potentially triggering effects.

“"I'm rushing through this because I already saw the heading ‘Case Studies’, and now I'm excited again. Because it’s a lot easier to read about someone else first, rather than telling so many things about yourself. And I think it’s so helpful when you can read about other people’s feelings, or experiences, because you see parallels and you immediately know ‘I’m not alone.’" (S2)

### Self-reflection exercises

Participants generally liked the self-reflection exercises as they delivered the content in an interactive manner, increased awareness of one’s feelings, and encouraged self-reflection. However, especially the self-reflection exercises had distressing effects when too demanding or when predefined answers mismatched with the personal experience of participants. Thus, participants stressed the need for clear instructions and optional assistance. Further, predefined answers should be differentiated and there should be an option for a free text answer.

“It’s good to not just read something or look at something, but to start reflecting about my own relationship.” (S6)

“It would be helpful […] to integrate exercises. So that the person in front of the computer knows, that they need to take some time to work through this. So that they don’t just rush through it and expect miracles.” (S4)

### Practical tips

Participants requested practical tips, which they could apply to and practice within their day-to-day life. The tips should provide practical suggestions addressing areas of difficulty, particularly to manage psychological distress and interpersonal conflicts.

“I thought this was great, because it’s something active, what they can take in their day-to-day life. Because that was my idea, that it would be nice if they would have something, which they can take to their day-to-day life and practice it.” (E1)

### Guidance

People with lived experiences mentioned the need for guidance to get support when the content was too distressing or demanding.

“It would need a contact person in the background. So that you have to possibility to say, oh, it triggered something, which I can’t handle any more right now. Then you’d need a real contact person, I guess.” (S4)

### Topics

We provide a list of the specific contents required in etable 2.

**eTable 2** Content considered relevant by the participants

| Topic | Specific content requested |
| --- | --- |
| Basic Information on IPV | - Definition - Different forms of IPV - Cycle of violence |
| Safety | - Danger Assessment - Safety-related behavior - Internet safety |
| Separation | - Decisional conflict - Preparing a separation |
| Children | - Instrumentalizing children in the context IPV - Consequences for children - Custody dispute |
| Help-seeking | - Help options - Step-by-step-instructions - Negative experiences with support services - Speaking about IPV experiences |
| Mental health | - Information on psychological consequences of IPV - Symptom check - Treatment options - Relevance of treatment - Mental health related stigma - Specific information (suicidality, alcohol and drug use, sleep, panic attacks) |
| Emotion regulation | - Relaxation/skills - Mindfulness - Anger and hate - Grief and loss - Guilt and shame |
| Self-esteem | - Influences - Associations with IPV - Instructions for self-care and positive self-talk |
| Posttraumatic growth | - Realizing personal strengths, resources and chances |
| Relationship | - Healthy and positive relationships - Needs and boundaries - Sexuality - Consent |

## Supplement D: Required design features

**eTable 3** Identified design features and illustrating quotes

| Functionality |  |
| --- | --- |
| Self-determination | “[…] they should have to option to choose: at this point, I don’t want to go any further. That’s too much right now, I’m not feeling well today, and then you can come back the next day or the next week and go on.” (S4)  “So that within the module, you have the choice, if you want to deal with these feelings, or if you want to leave them aside right now.” (S1)  “The possibility to get in-depth information, yes or no, it offers me the freedom to choose” (S3) |
| Individualized | “When they answer like this, they will get this, when they answer like that, they will get that.” (S4) |
| Interactive | “Oh, I answer the question how I’m doing and I get a reaction, that’s nice.” (S5) “  Wow, that’s quite a bit of text. […] It would be cool if it would be more interactive here.” (S2) |
| Multimodality | “I really appreciated that I could listen to and read the text at the same time” (E5)  “It’s nice that there is also a text. […], because of my alertness I have a hard time listening to something. But this way I can just read through it in peace.” (S2) |
| Easy handling & clear instructions | “I don’t know if I would have noticed that I need to click here.” (S2) |
| Security measures | “This exit button and the possibility, that you can’t track on your chronic, where [which website] I’ve been to.” (E5) |
| Aesthetic |  |
| Clearly arranged | “That’s good, this box, which summarizes the important aspects. And the color highlighting.” (E3) |
| Neutral | “I really liked that the design was simple” (S1) |
| Calming | “I think when you’re so distressed, you prefer something that’s calming […] like this picture with the water.”(S6) |
| Feel-good atmosphere | “To create feel-good-atmosphere for those people, who don’t feel well, so that it will be easier for them to focus on positive aspects.” (S5) |
| Pictures | “Somehow I feel like that’s a picture from an advertisement. It doesn’t look like a professional support service.” (S6) |
| Structure |  |
| Short | “More is less! Less text, less exercises, less of everything.” (S5) |
| Logical | “I thought the structure was very good, logical and clear.” (E4) |
| Predictability | “I liked the preview with the content of the module. […] So that you know, what comes next.” (S5) |
| Gentle start | “To start with self-reflection exercises, it’s deep. […] I prefer to read about others first.” (S2) |
| Language |  |
| Plain | “I think, the only thing I didn’t like was the language, it was too complicated. […] I think you can dare to use really simple language.” (E1) |
| Direct | “That’s such an important message. I wonder whether you could address the reader more directly here.” (E1) |
| Personal | “I think the text was a little to impersonal. A little too scientific” (S1) |
| Appreciative | “I think, a lot of clients would feel welcomed, […] and seen.” (E1) |
| Differentiated | “This kind of wording, I find a little difficult, because it generalizes.” (S4) |
| Positive | “I think I’d frame this a little more positive.” (S3) |
| Sensitive | “Give this advice carefully and sensitive.” (S4) |
| Metaphors and analogies | “I think, these metaphors, they are so strong. I think it really clicks with clients.” (E1) |
| Inclusiveness & Diversity |  |
| Gender | “I think a lot of men, too, are affected.” (S1) |
| Sexuality | “It’s a little stigmatizing. Even if it’s mostly men, women, too, can be perpetrators.” (E2) |
| Culture | “[…] A lot of women or men, [they have] other life realities. First, it’s two German names, both are white, University, the case examples could be a little more diverse in regard to the life realities.” (E4) |
| Education |  |
| Accessibility |  |
| Professional appearance | “I think if it says something like ‘University of Freiburg’, then I’d trust it.” (S1) |
| Public advertisement & Personal recommendation | “If my GP would tell me to look at this, would give me a flyer, and would say that maybe this could support me, I think then I’d use it.” (E3)  “One could recommend this website. I think it’s better than a telephone number. You hope that, in a private moment, they will open it.” (S5)  “I don’t know if my patients would google ‘violence’, probably, maybe ‘violence support’, then it would need to show up. And then I think […] the landing page should be very inviting, should offer an explanation why it could be helpful.” (E1)  “A lot of information material, flyer, you need to advertise it, with cooperating institutions, counseling services […]” (E4) |
| Anonymity | “I think, to some extent, anonymity would be important. [….]. You know, there is a lot of shame to confess, that you use such a program.” (E2) |
| Low cost | “Of course it would matter, if it is for free, or how much it would cost.” (S2) |
| Smartphone option | “I seldom use my PC, when I’m in the internet, I use my phone” (S2) |
